# Insights into the binding and covalent inhibition mechanism of PF-07321332 to SARS-CoV-2 M^pro^†

**DOI:** 10.1039/d1ra08752e

**Published:** 2022-01-28

**Authors:** Son Tung Ngo, Trung Hai Nguyen, Nguyen Thanh Tung, Binh Khanh Mai

**Affiliations:** Laboratory of Theoretical and Computational Biophysics, Ton Duc Thang University Ho Chi Minh City Vietnam ngosontung@tdtu.edu.vn; Faculty of Applied Sciences, Ton Duc Thang University Ho Chi Minh City Vietnam; Institute of Materials Science, Vietnam Academy of Science and Technology Hanoi 11307 Vietnam; Graduate University of Science and Technology, Vietnam Academy of Science and Technology Hanoi 11307 Vietnam; Department of Chemistry, University of Pittsburgh Pittsburgh PA 15260 USA binh.mai@pitt.edu

## Abstract

The severe acute respiratory syndrome coronavirus 2 (SARS-CoV-2) has been causing the COVID-19 pandemic, resulting in several million deaths being reported. Numerous investigations have been carried out to discover a compound that can inhibit the biological activity of the SARS-CoV-2 main protease, which is an enzyme related to the viral replication. Among these, PF-07321332 (Nirmatrelvir) is currently under clinical trials for COVID-19 therapy. Therefore, in this work, atomistic and electronic simulations were performed to unravel the binding and covalent inhibition mechanism of the compound to M^pro^. Initially, 5 μs of steered-molecular dynamics simulations were carried out to evaluate the ligand-binding process to SARS-CoV-2 M^pro^. The successfully generated *bound* state between the two molecules showed the important role of the PF-07321332 pyrrolidinyl group and the residues Glu166 and Gln189 in the ligand-binding process. Moreover, from the MD-refined structure, quantum mechanics/molecular mechanics (QM/MM) calculations were carried out to unravel the reaction mechanism for the formation of the thioimidate product from SARS-CoV-2 M^pro^ and the PF-07321332 inhibitor. We found that the catalytic triad Cys145–His41–Asp187 of SARS-CoV-2 M^pro^ plays an important role in the activation of the PF-07321332 covalent inhibitor, which renders the deprotonation of Cys145 and, thus, facilitates further reaction. Our results are definitely beneficial for a better understanding of the inhibition mechanism and designing new effective inhibitors for SARS-CoV-2 M^pro^.

## Abstract 2

The catalytic triad Cys145–His41–Asp187 of SARS-CoV-2 M^pro^ plays an important role in the activation of the PF-07321332 covalent inhibitor.

## Graphical abstract

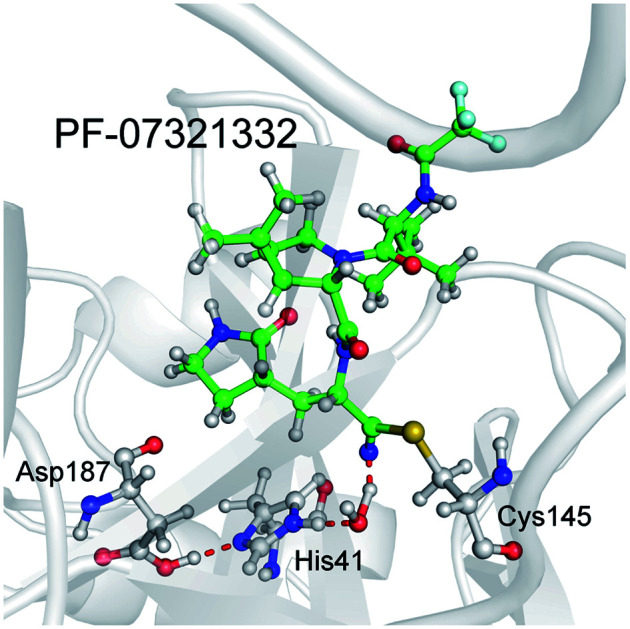

## Introduction

The severe acute respiratory syndrome coronavirus 2 (SARS-CoV-2), a β-coronavirus belonging to the Coronaviridae virus family, has caused the global pandemic named coronavirus disease 2019 (COVID-19).^1–3^ SARS-CoV-2, which was thought to originate from bats, can rapidly transfect between humans and humans.^4^ The virus can be rapidly spread among the community *via* aerosol transmission.^5,6^ Despite international exertions to restrict the rate of the virus spreading, the number of infected cases has increased.^7^ Moreover, although three vaccines, the Pfizer-BioNTech, Moderna, and Janssen COVID-19 vaccines, have been approved by the FDA for emergency use,^8^ the pandemic is still causing numerous issues to community health. In particular, recent work has suggested that long-term health problems of fully recovered patients with no or minor symptoms are increasingly being recorded.^9^ Furthermore, a growing number of variants escaping from the neutralizing antibodies have been observed.^10,11^ These variants contain mutations in the piece of the genome encoding the spike protein, which has been used by the vaccines to generate immunity.^12,13^ The vaccine effectiveness in the near future will likely be decreased. Therefore, developing an appropriate treatment for COVID-19 is accordingly of great urgency.

The viral genome, with a length of 29.2 kb, encodes more than 20 nonstructural (nsp) and structural proteins.^1,14^ Those of SARS-CoV and SARS-CoV-2 are more than 82% similar to each other.^15^ In particular, SARS-CoV-2 consists of two proteases, the SARS-CoV-2 main protease (M^pro^ or 3CL^pro^) and papain-like protease (PL^pro^), which correspond to nsp5 and nsp3, respectively. The main protease (M^pro^) of SARS-CoV-2 virus has >96% sequence identity to the one of SARS-CoV,^16,17^ while the SARS-CoV-2 PL^pro^ shares 83% sequence identity to the SARS-CoV PL^pro^.^18^ The protease first self-cleaves from the product of the messenger ribonucleic acid (mRNA) translation, and polyproteins are then cleaved to polypeptides. Because the polypeptides are required for viral replication and encapsulation, the proteases are directly associated with viral replication and proliferation.^16,17^ In more detail, PL^pro^ responds to the formation of nsp1-3 and M^pro^ is required for the establishment of the nsp4-16.^19^ Therefore, the SARS-CoV-2 M^pro^ has become a high-profile target for antiviral drug design, since inhibiting the biological activity of the SARS-CoV-2 M^pro^ is able to prevent the replication of a new virus.

Although there are already positive signs in the development of COVID-19 therapies,^20^ the race for antiviral drugs to prevent COVID-19 continues to be urgent.^21^ Numerous investigations have thus been performed to characterize a potential inhibitor for the SARS-CoV-2 M^pro^.^22–36^ Several compounds have been suggested to be able to inhibit the biological activity of M^pro^. In this context, a compound, named PF-07321332, has emerged as one of the most potent candidates for an oral antiviral therapeutic factor. The compound is currently under clinical trials as an antiviral agent against SARS-CoV-2 and was shown to be a potential inhibitor for M^pro^ in *in vitro* studies^37,38^ and phase I clinical trials.^39^ Moreover, a clinical phase III study in non-hospitalized high-risk adults with COVID-19 has also started.^40^ Understanding the binding and covalent inhibition mechanism of PF-07321332 to the SARS-CoV-2 M^pro^ would be beneficial in the design of antivirus drugs. Therefore, in this work, we tried to reveal physical insights into the binding and covalent inhibition mechanism of PF-07321332 to the SARS-CoV-2 M^pro^. The work was supported by steered-molecular dynamics (SMD) and quantum mechanics/molecular mechanics (QM/MM) simulations. In particular, SMD simulations were first employed to preliminarily evaluate the binding pose of the ligand to M^pro^. QM/MM calculations then probed the covalent inhibition mechanism. The obtained results are believed to enhance COVID-19 therapy.

## Computational methods

### Structures of the receptor and ligand

**Fig. 1:**
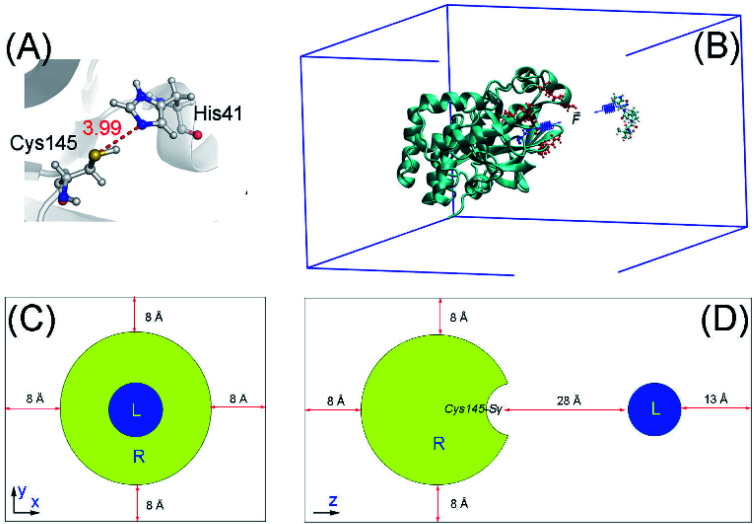
(A) The protonation states of the catalytic dyad. The distance between the Cys145-Sγ and His41-Nε atoms from the X-ray diffraction structure is also presented. (B) Three-dimensional structure of the SARS-CoV-2 M^pro^ (PDB ID 7JYC) + PF-07321332. (C) and (D) System configuration. In particular, the minimum distance between SARS-CoV-2 M^pro^ Cys145-Sγ and the ligand is *ca.* 28 Å. A constant force with a spring constant cantilever of 1 kcal mol^−1^ Å^−2^ was put on SARS-CoV-2 M^pro^ Cys145-Sγ and the nitrile group of the ligand. The solvent was hidden to clarify the view.

The three-dimensional conformation of the SARS-CoV-2 M^pro^ was downloaded from the Protein Data Bank (PDB) with the identity 7JYC.^41^ The protonation states of the M^pro^ catalytic dyad, including His41 and Cys145, were assigned, as shown in Fig. 1A, since it plays an important role in the protease activity and ligand effectiveness.^42^ The three-dimensional structure of PF-07321332 was generated using MarvinSketch, a package of ChemAxon.^43^ The ligand structure was then optimized *via* density-functional theory (DFT) calculations with the B3LYP functional at the 6-31G(d,p) level of theory. During QM calculations to prepare the ligand for MD simulations, the implicit solvent environment, *ε* = 78.4, was implemented.

### Atomistic simulations

Atomistic simulations were performed to obtain the binding pose between SARS-CoV-2 M^pro^ + PF-07321332 using GROMACS version 2019.^44^ The protease and ions were presented using the Amber14SB force field.^45^ The TIP3P water model was employed for water molecules.^46^ PF-07321332 was topologized *via* the general Amber force field^47^ with the support of the AmberTools18 and ACPYPE packages.^48,49^ In particular, the QM calculations using the B3LYP functional at the 6-31G(d,p) level of theory with the implicit solvent were performed to obtain the ligand geometrical information and atomic charges. The restrained electrostatic potential scheme was employed to estimate the ligand atomic charges.^47^ In particular, the ligand was placed on the position having a minimum distance from the Cys145-Sγ atom of 28 Å, as shown in Fig. 1. The complex was then inserted into a rectangular periodic boundary condition (PBC) box with a size of 9.40 × 5.65 × 8.51 nm^3^, as shown in Fig. 1. The soluble complex contained 43 789 atoms, involving the protease, PF-07321332, 13 012 water molecules, and 4 Na^+^ ions.

Atomistic simulations were carried out with the parameters referred to in the previous studies.^50,51^ The simulations were executed at 310 K. A non-bonded contact between two atoms is available when the pair distance is smaller than 0.9 nm. The electrostatic (cou) and van der Waals (vdW) interactions were computed using the fast particle-mesh Ewald electrostatics and cut-off approaches, respectively.^52^ The solvated system was first minimized using the steepest descent method. The minimized system was then relaxed using NVT and NPT simulations with a length of 0.1 ns each. During these simulations, the SARS-CoV-2 M^pro^ C_α_ atoms and the ligand atoms were positionally restrained *via* a small harmonic force having *ca.* 24 kcal mol^−1^ nm^−2^ spring constant.

### Steered-molecular dynamics (SMD) simulations

The last conformation of NPT simulations was then used as the starting shape for further SMD simulations. During the SMD simulating process, a small constant force with a spring constant of 1 kcal mol^−1^ nm^−2^ was employed to pull the nitrile group of PF-07321332 and the sulfur atom of the residue Cys145 together because a covalent bond is able to form between the two groups.^53^ During the SMD simulations, the SARS-CoV-2 M^pro^ reorientation and translation were prevented *via* a small restraining force applied on the C_α_ atoms. The ligand was slowly mobilized from the *unbound* to the *bound* state under the effects of a small constant force, with a schematic representation of the simulations shown in Fig. 1. It should be noted that using a stronger pulling force can make the ligand bind quicker, but at the same time may cause distortion to the binding pocket and result in the ligand adopting the wrong binding pose. We found that the chosen pulling strength is a good trade off between computational efficiency and accuracy. Moreover, because the pulling force is very weak, the ligand would mobilize very slowly and probably fail to bind to the active site of the protease. SMD simulations were thus carried out with a length of 50 ns and repeated 81 times independently to produce the binding conformation of SARS-CoV-2 M^pro^ + PF-07321332. In addition, a restraining force with *ca.* 24 kcal mol^−1^ nm^−2^ spring constant was also applied on SARS-CoV-2 M^pro^ C_α_ atoms to avoid system reorientation. Furthermore, the trajectory was extended to 1.0 μs of MD simulations to allow the system enough time to reach the “native” binding pose. There were 5 μs of MD simulations in total, which were produced to assess the binding process of PF-07321332 to the SARS-CoV-2 M^pro^. The coordinates of the complex were recorded every 1 ps.

### QM/MM calculations

**Scheme 1:**
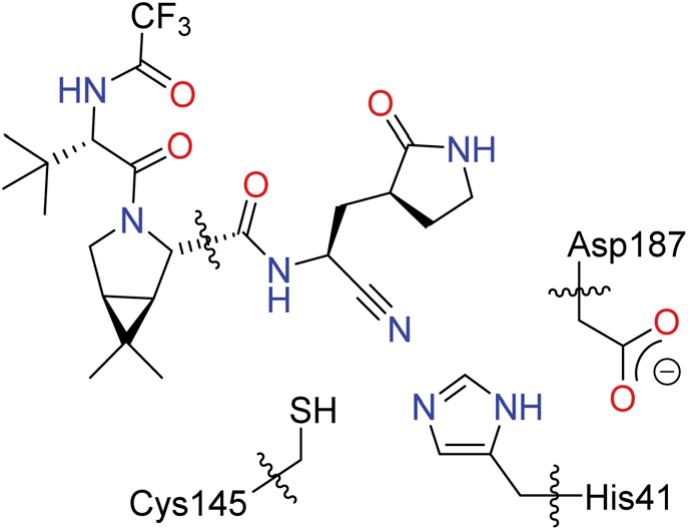
The definition of the QM region for the ONIOM calculations.

The covalent inhibition mechanism for the reaction between PF-07321332 and the SARS-CoV-2 M^pro^ to give the thioimidate product was investigated using the ONIOM algorithm^54^ implemented in Gaussian 16.^55^ The MolUP package^56^ was used to support input preparations. A representative snapshot of the SARS-CoV-2 M^pro^ + PF-07321332 complex in the minimum region of the free energy landscape was used as the starting structure for the ONIOM calculations (see the Results and discussion section). Only waters and counter-ions within a distance of 7 Å from the protein were kept, giving a system with a total of 16 327 atoms and a neutral charge. The MM parameters and atomic charges were extracted from the parameter from MD simulations (*vide supra*). The QM region for the ONIOM calculations was defined as shown in Scheme 1, including Cys145, His41, and Asp187 residues. To facilitate the calculations, only the part of the PF-07321332 that is close to Cys145 is included in the QM region (Scheme 1). Hydrogen-link (H-link) atoms were added to the QM atoms at the boundary. The QM region has 49 atoms, including H-link atoms, with a total charge of −1.

All intermediates and transition states were fully optimized using the quadratic coupled algorithm^57^ with the dispersion-corrected^58^ B3LYP functional,^59,60^*i.e.*, the B3LYP-D3(BJ), and 6-31G(d) basis sets. All residues in the MM region which were not within 10 Å of the QM region were constrained in all calculations. Vibrational frequency calculations at the same level of theory as the optimization were performed to confirm if each structure was a local minimum (no imaginary frequency) or a transition state (one imaginary frequency). Single-point calculations were carried out using the M06-2X functional^61^ and 6-311+G(2d,2p) basis set. The free energy profile was constructed by the Gibbs free energy differences between stationary points, *i.e.*, intermediates and transition states, and the SARS-CoV-2 M^pro^–PF-07321332 complex.

### Analysis tools

The collective variable free energy landscape (FEL) was constructed using “gmx sham”, a tool of GROMACS. The two variables were the non-hydrogen atom root-mean-square deviation (RMSD) of the complex and the distance between the sulfur atom of the Cys145 residue and the nitrile group of PF-07321332. All of the snapshots locating the minima were used as the initials of the clustering analysis. The clustering method was applied with a non-hydrogen RMSD of nine critical residues of the SARS-CoV-2 M^pro^ and the ligand PF-07321332. Specifically, the nine critical residues were Thr26, His41, Ser46, Asn142, Gly143, Cys145, His164, Glu166, and Gln189, which play an important role in the ligand-binding process of SARS-CoV-2 M^pro^.^62^ The clustering cutoff was 1 Å. The protonation states of PF-07321332 were predicted using Chemicalize.^63^

## Results and discussion

In this work, SMD simulations were employed to search for the binding position between PF-07321332 and the SARS-CoV-2 M^pro^. In particular, the nitrile group of PF-07321332 and the sulfur atom of the Cys145 residue were pulled together using a small constant force. 81 SMD trajectories with a length of 50 ns each were produced to investigate the diffusion of PF-07321332 around the SARS-CoV-2 M^pro^ active site. Among these, 21 trajectories (26%) successfully reached the binding pocket, since the distances between Cys145-Sγ and the non-hydrogen atoms of PF-0321332 were less than 4.5 Å (Fig. S1–S9 of the ESI).† However, there were only 13 trajectories (16%), where the ligand was stable in the binding pocket until the trajectories were completed. Some trajectories reached the binding pocket just after *ca.* 4 ns of simulation, whereas some required more than *ca.* 40 ns.

**Fig. 2:**
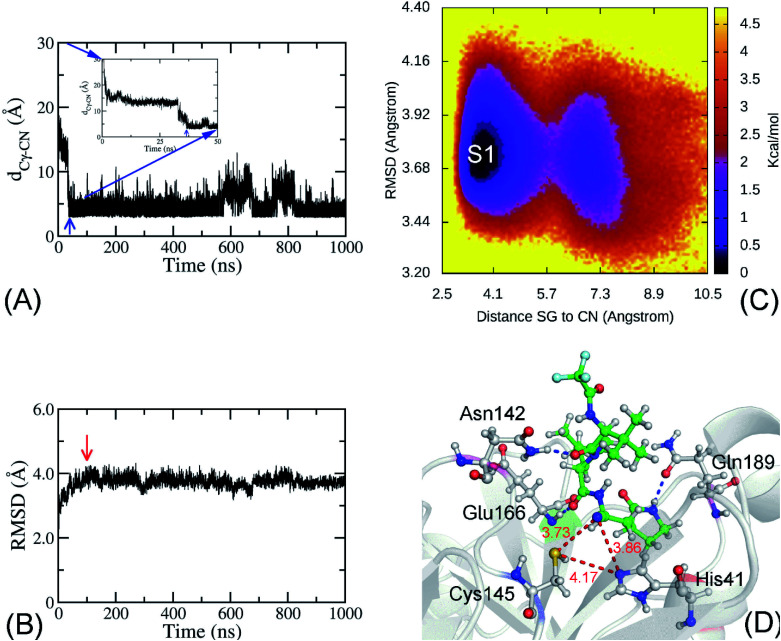
(A) The time dependence of the distance *d*_Sγ–CN_. The blue arrow indicates when the ligand PF-07321332 reaches the *bound* state at *ca.* 36.5 ns. The inset is a zoomed in section of the figure over the interval 0–50 ns. (B) Non-hydrogen RMSD of the complex SARS-CoV-2 M^pro^ + PF-07321332 over SMD simulations. The red arrow indicates when the complex reaches the equilibrium state at *ca.* 100 ns. (C) The collective-variable FEL was constructed over the equilibrium interval 100–1000 ns. The two reaction coordinates were the non-hydrogen RMSD of the complex and the distance *d*_Sγ–CN_. The minimum, denoted as S1, is at (*d*_Sγ–CN_, RMSD) coordinates of (3.74, 3.72). (D) is the representative structure of the complex, which corresponds to the minimum S1. In particular, the ligand formed HBs to residue Asn142, Glu166, and Q189 (the blue dashed line). The red dashed lines and red numbers denote the distances between two atoms in Å.

Among the 21 trajectories mentioned above, 10 of them (12%) successfully generated the *bound* states by forming a contact between the Cys145-Sγ atom and the nitrile group of PF-07321332, in which the distance (*d*_Sγ–CN_) was less than 4.5 Å (Fig. 2A and S9–S17 of the ESI).† It should be noted that a short contact between two groups would allow the nitrile group and the catalytic cysteine to be able to adopt a covalent bond between them.^53^ PF-07321332 required more time (at least *ca.* 4.5 ns) to reach the *bound* state after entering the binding pocket of the SARS-CoV-2 M^pro^. However, there were only 2 trajectories where the *d*_Sγ–CN_ stayed below 4.5 Å until the simulations were completed (Movie S1† describes a representative binding process). Moreover, although the binding mechanism of PF-07321332 probably is a complex pathway instead of a simple mobilization of the ligand to the pocket,^64^ two successfully generated *bound* states trajectories preliminarily suggested that the residues Glu166 and Gln189 play an important role during the binding process of the inhibitor (Fig. S18 and S19 of the ESI).† Furthermore, analyzing these two trajectories indicated that the pyrrolidinyl group of the ligand first inserted itself into the space between the residues Glu166 and Gln189, before the whole of PF-07321332 fully inserted into the binding pocket of the SARS-CoV-2 M^pro^.

Because the nitrile group of PF-07321332 likely forms a covalent bond with the Cys145-Sγ atom, in which the force constant is much larger than 1 kcal mol^−1^ nm^−2^, one of the successfully generated *bound* state trajectories was extended to 1 μs to assess the stability of the complex. The non-hydrogen RMSD of the complex reaches a stable state after *ca.* 100 ns of SMD simulations (Fig. 2B). 900 000 snapshots over the interval 100–1000 ns were collected to be inputs for the collective-variable FEL analysis. The non-hydrogen RMSD of the complex and the distance *d*_Sγ–CN_ were calculated over these conformations to use as the two reaction coordinates of FEL. The obtained FEL are shown in Fig. 2C, with one minimum denoted as S1. The binding pose of PF-0721332 to the SARS-CoV-2 M^pro^ in the representative structure S1 is shown in Fig. 2D. In particular, the ligand nitrile group formed a contact with the Cys145-Sγ atom at a distance of *d*_Sγ–CN_ = 3.73 Å. The ligand nitrile group also adopted a contact with the His-Nε atom with *d*_Nε–CN_ = 3.86 Å. The presence of contacts between the PF-0721332 nitrile group increases the distance between the Cys145-Sγ and His41-Eε atoms, *d*_Nε–Sγ_, from 3.99 to 4.17 Å (Fig. 1A and 2D). Therefore, the catalytic dyad Cys145–His41 is probably disturbed. In addition, the ligand also formed HBs to three residues, Asn142, Glu166, and Gln189 (Fig. 2D).

**Scheme 2:**
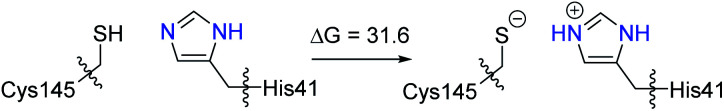
Computed free energy difference (kcal mol^−1^) for the ion pair formation of the catalytic dyad (Cys145^−^–His41H^+^) from the neutral form (Cys145H–His41).

In recent theoretical studies,^42,65–68^ using the adaptive string method, Moliner and Tuñón *et al.* proposed that the catalytic dyad Cys145H–His41 plays an important role in the reactivity of the SARS-CoV-2 M^pro^, where a proton transfer from Cys145H to His41 takes place, first giving the ion pair Cys145^−^–His41H^+^, which is followed by a nucleophilic addition, producing a covalent bond with the inhibitor. In our initial ONIOM calculations, only the residues Cys145 and His41, and the PF-0721332 inhibitor were included in the QM region. However, we were unsuccessful in locating the ion pair Cys145^−^–His41H^+^. During the optimization, the proton automatically transferred from His41H^+^ to Cys145^−^ (see Fig. S20 of the ESI).† We then performed a constrained optimization by fixing the N–H distances to 1.01 Å. Interestingly, the ion pair structure was calculated to be 31.6 kcal mol^−1^ higher in energy than the neutral form (Scheme 2). Our DFT calculations suggest that the imidazole ring of His41 is not basic enough to abstract a proton from the thiol group of Cys145. Our QM/MM calculations are consistent with a recent study, where the ion pair of the catalytic dyad (Cys145^−^–His41H^+^) was suggested to be a transient intermediate and was very high in energy compared to the neutral form.^69^

**Fig. 3:**
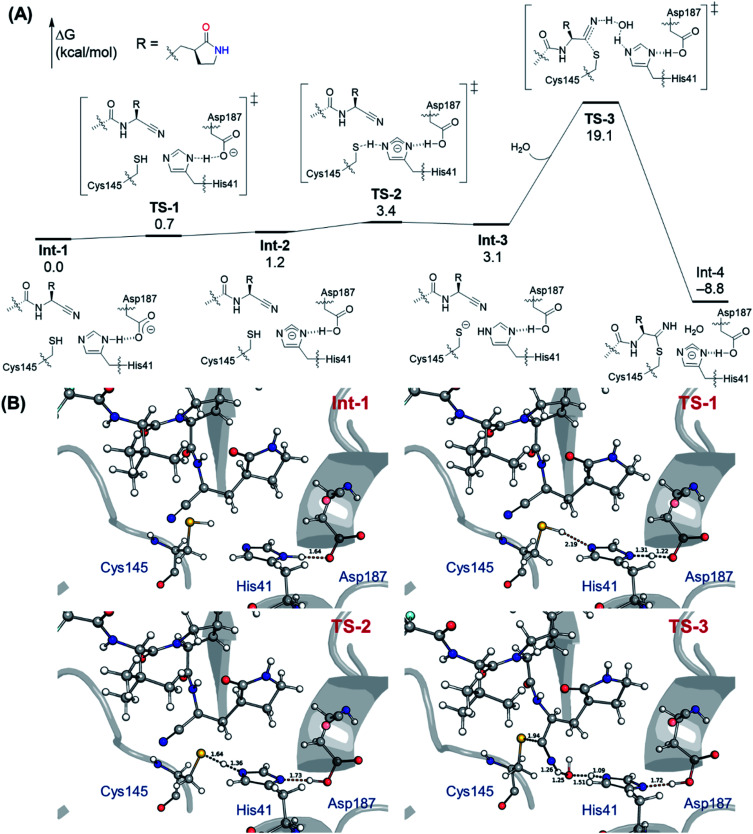
Computed free energy profile (in kcal mol^−1^) and optimized structures of the reactant complex and transition states for the covalent inhibition mechanism of PF-07321332 with the SARS-CoV-2 M^pro^. All distances are given in Å.

Searching for alternative mechanisms for this reaction, we found that the residue Asp187 was close to the residue His41, which could form a catalytic triad,^70^ Cys145–His41–Asp187, which facilitated the deprotonation of Cys145. Therefore, the Asp187 residue was then included in our ONIOM calculations. The computed free energy profile for the covalent inhibition mechanism between the SARS-CoV-2 M^pro^ and PF-0721332 is shown in Fig. 3. At Int-1, a strong hydrogen bond with a distance of 1.64 Å between His41 and Asp187 is found. A proton transfer *via*TS-1 between His41 and Asp187 can easily take place, giving His41^−^, which is followed by another proton transfer *via*TS-2 from Cys145 to His41^−^, generating Cys145^−^. The activation barriers of the two proton transfer steps, TS-1 and TS-2, are very low, amounting to 0.7 and 3.4 kcal mol^−1^, respectively, relative to Int-1. It should be noted that although the electronic energy of TS-1 is calculated to be 0.5 kcal mol^−1^ higher than that of Int-2, because of the entropic effect, the Gibbs free energy value of TS-1 is slightly lower than that of Int-2 (Fig. 3).

From Int-2, the nucleophilic addition of Cys145^−^ to the nitrile group of PF-07321332 *via*TS-3 can then take place. Interestingly, at TS-3, a water molecule is crucial to stabilize the developed negative charge on the nitrogen atom of the nitrile group resulting from the nucleophilic addition of Cys145^−^. The small size of the nitrile group allows for the water molecule to participate in the reaction and transfer proton from His47 to nitrogen atom. In the nucleophilic addition TS-3, water played an essential role, where it stabilized the developed negative charge on the nitrogen atom of the nitrile group and, thus, facilitated this step.^42,65–68^ We also tried to optimize the nucleophilic addition without a water molecule. However, no TS could be located. The activation barrier of TS-3 was calculated to be 19.1 kcal mol^−1^ relative to Int-1, which is consistent with the previous study by Tuñón and co-workers.^42^ Our reaction mechanism is in good agreement with the covalent inhibition mechanism of antidiabetic drugs in dipeptidyl peptidase-4.^71^ Furthermore, the activation barrier of 19.1 kcal mol^−1^ of this reaction is also consistent with a previous study, where energy barriers of enzymatic reactions were found to be in a range of 14–20 kcal mol^−1^.^72^

It should be mentioned that from Int-4, the hydrolysis of the S–C bond could also occur by the nucleophilic addition of water to form an amide product. (Fig. S21 of the ESI† for the optimized transition state). However, the activation barrier of this transition state was calculated to be 39.6 kcal mol^−1^ relative to Int-3, ruling out this possibility and validating the inhibition ability of PF-07321332 to the SARS-CoV-2 M^pro^. Furthermore, we also optimized the alternate mechanism where the proton transfer and nucleophilic addition occur synchronously. However, no transition state for this mechanism could be located because of the instability of the high negative partial charge in the nitrogen atom of the nitrile group of PF-07321332.

Based on our ONIOM calculations, we found that the catalytic triad Cys145–His41–Asp187 plays an important role in the covalent inhibition of the SARS-CoV-2 M^pro^, which enables the deprotonation of Cys145 and, thus, facilitates further reaction. This finding is consistent with a previous study^73^ which demonstrated that Asp187 favors proton transfer from Cys145 to His41.

## Conclusions

In this work, 5 μs of SMD simulations were first generated to preliminarily estimate the binding pose of PF-07321332 to the SARS-CoV-2 M^pro^. In particular, the ligand reached the binding pocket of SARS-CoV-2 M^pro^ in 26% of the trajectories. Among these, the nitrile group of PF-07321332 successfully adopted a contact with the Cys145-Sγ atom in 12% of the trajectories. However, the contact only stabilized over 2 trajectories until the simulations were completed. Moreover, the residues Glu166 and Gln189 were suggested to play an important role during the binding process of the inhibitor. The pyrrolidinyl group of PF-07321332 is probably key in leading the compound into a successful *bound* state.

A representative structure of SARS-CoV-2 M^pro^ + PF-07321332 was obtained by using a combined calculation of FEL and clustering analyses. In this state, the distance between the Cys145-Sγ and His41-Eε atoms *d*_Nε–Sγ_ was increased from 3.99 to 4.17 Å when the PF-07321332 nitrile group adopted a contact with the Cys145-Sγ atom (*d*_Sγ–CN_ = 3.73 Å). The catalytic dyad Cys145–His41 is probably disturbed. In addition, three residues, Asn142, Glu166, and Gln189, play a crucial role in the ligand-binding process by forming HBs with the inhibitor.

From the representative structure of the complex, quantum mechanics/molecular mechanics (QM/MM) calculations were performed to unravel the reaction mechanism for the formation of the thioimidate product from the SARS-CoV-2 M^pro^ and the PF-07321332 inhibitor. We found that the catalytic triad Cys145–His41–Asp187 of the SARS-CoV-2 M^pro^ plays an important role in the activation of the PF-07321332 covalent inhibitor, which leads to the deprotonation of Cys145 and, thus, facilitates further reaction. The outcome is in good agreement with a previous study^73^ which found that Asp187 favors proton transfer from Cys145 to His41. Our results are definitely beneficial for a better understanding of the inhibition mechanism and designing new effective inhibitors for the SARS-CoV-2 M^pro^.

## Conflicts of interest

There are no conflicts to declare.

## Supplementary Material

RA-012-D1RA08752E-s001

RA-012-D1RA08752E-s002
