# Supplementary material for: Insights into the binding and covalent inhibition mechanism of PF-07321332 to SARS-CoV-2 Mpro
Source: RSC Adv. 2022 Jan 28;12(6):3729–37. doi: 10.1039/d1ra08752e (PMC8979274; doi:10.1039/d1ra08752e)
Supplement: RA-012-D1RA08752E-s002 [file RA-012-D1RA08752E-s002.pdf]

Electronic Supplementary Material (ESI) for RSC Advances.
This journal is © The Royal Society of Chemistry 2022

Supporting information

Insights into the Binding and Covalent Inhibition Mechanism of PF-07321332 to SARS-CoV2 Mpro

Son Tung Ngo,[a,b,*] Trung Hai Nguyen,[a,b] Nguyen Thanh Tung,[c,d] and Binh Khanh Mai[e,*]

[a]*Laboratory of Theoretical and Computational Biophysics, Ton Duc Thang University, Ho Chi Minh City 72915, Vietnam*

[b]*Faculty of Applied Sciences, Ton Duc Thang University, Ho Chi Minh City 72915, Vietnam*

[c]*Institute of Materials Science, Vietnam Academy of Science and Technology, 11307 Hanoi, Vietnam*

[d]*Graduate University of Science and Technology, Vietnam Academy of Science and Technology, 11307 Hanoi, Vietnam*

[e]*Department of Chemistry, University of Pittsburgh, Pittsburgh, PA, 15260, USA*

*Email: ngosontung@tdtu.edu.vn and binh.mai@pitt.edu

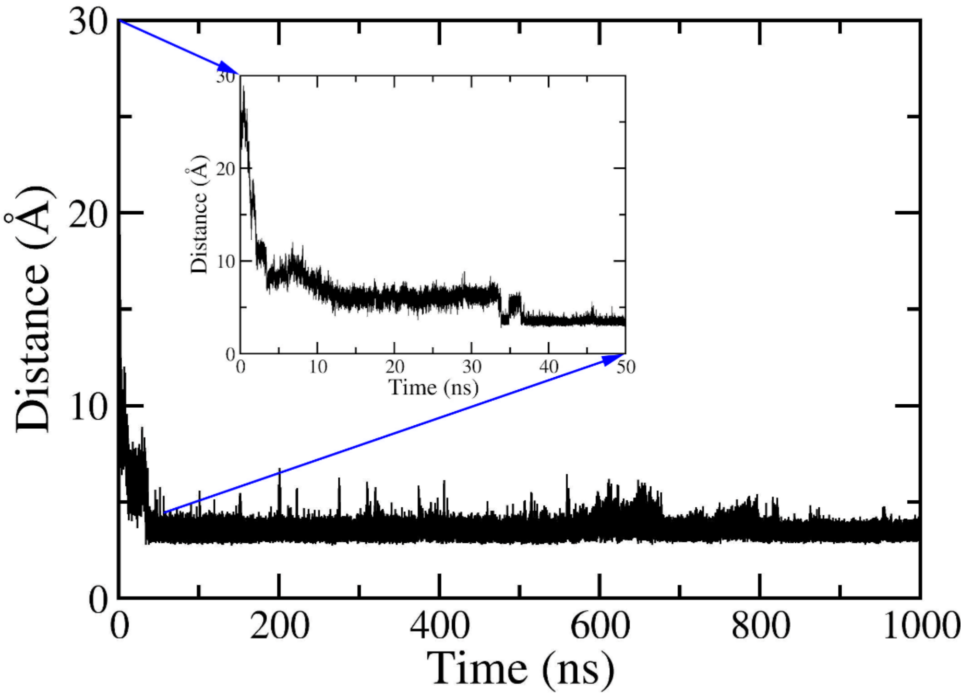

Figure S1. The minimum distance between non-hydrogen atoms of PF-07321332 and non-hydrogen atoms of the catalytic dyad (His41 and Cys145 residues). The results were obtained due to analyzing trajectory 0 over 50 ns (zoomed figure) and 1000 ns of SMD simulations. When the distance is smaller than 4.5 Å, the ligand reached the binding pocket of SARS-CoV-2 Mpro.

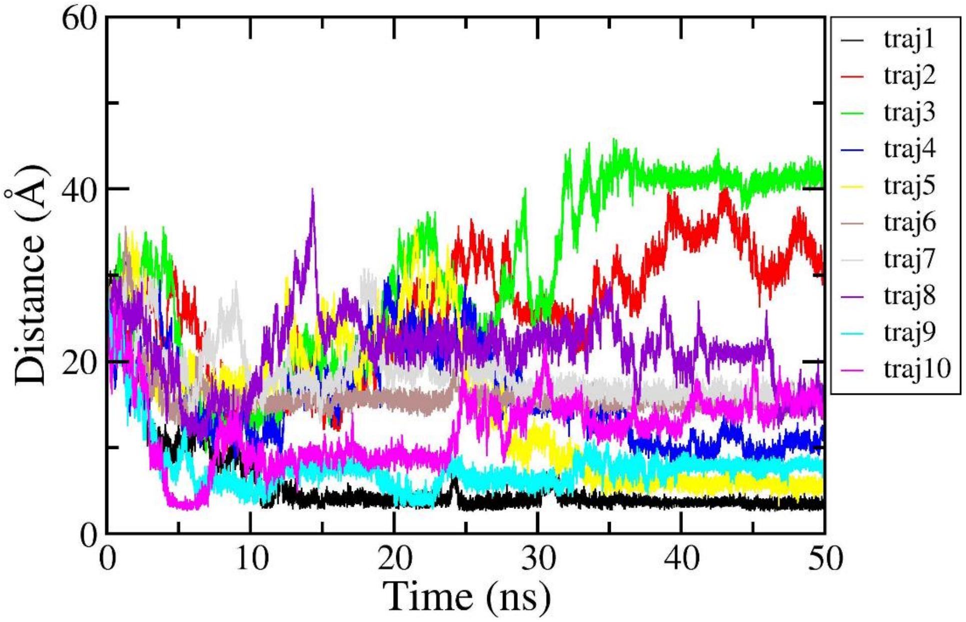

Figure S2. The minimum distance between non-hydrogen atoms of PF-07321332 and non-hydrogen atoms of the catalytic dyad (His41 and Cys145 residues). The results were obtained due to analyzing trajectories 1-10. When the distance is smaller than 4.5 Å, the ligand reached the binding pocket of SARS-CoV-2 Mpro.

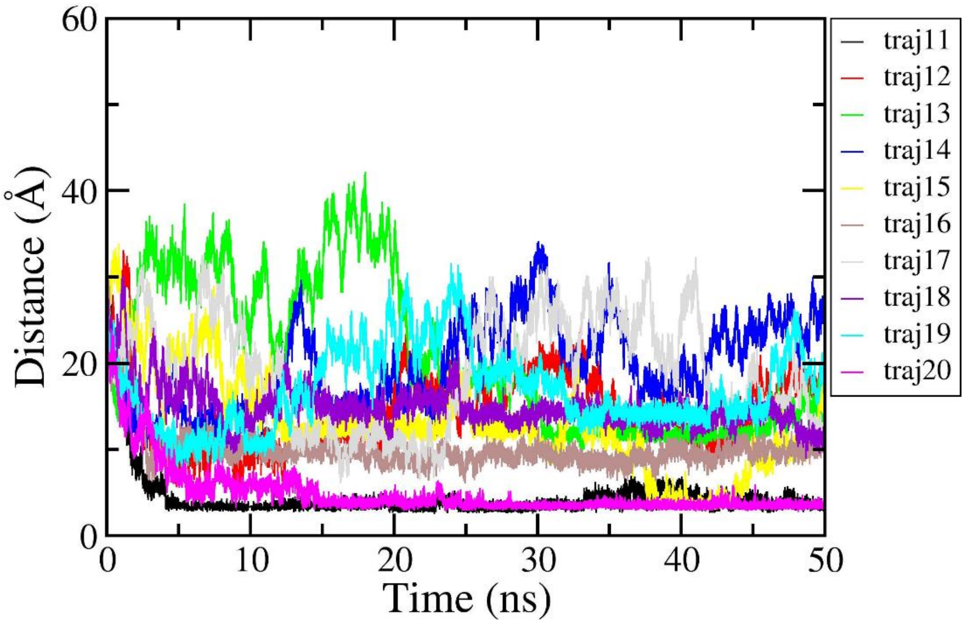

Figure S3. The minimum distance between non-hydrogen atoms of PF-07321332 and non-hydrogen atoms of the catalytic dyad (His41 and Cys145 residues). The results were obtained due to analyzing trajectories 11-20. When the distance is smaller than 4.5 Å, the ligand reached the binding pocket of SARS-CoV-2 Mpro.

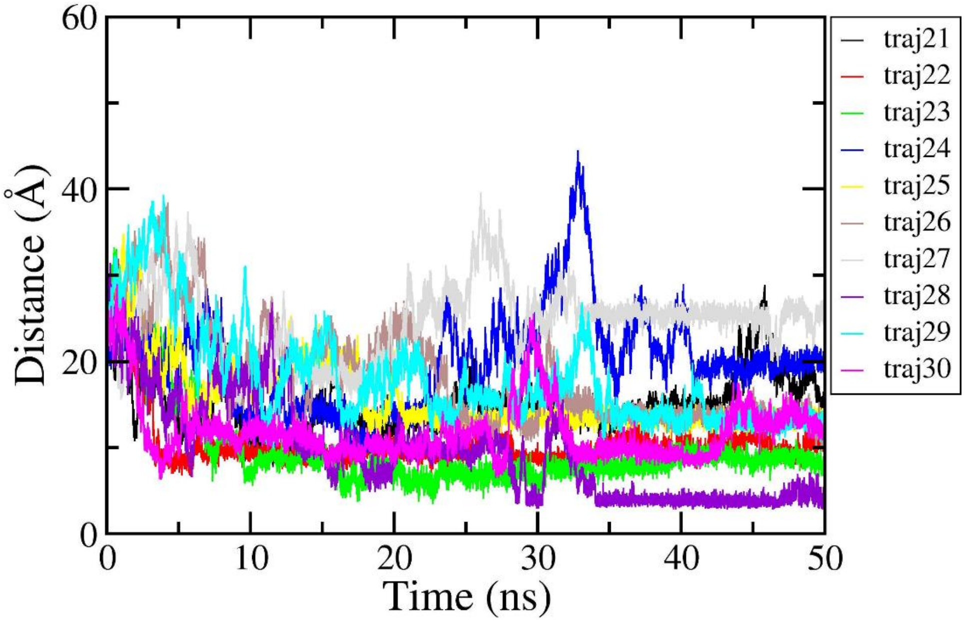

Figure S4. The minimum distance between non-hydrogen atoms of PF-07321332 and non-hydrogen atoms of the catalytic dyad (His41 and Cys145 residues). The results were obtained due to analyzing trajectories 21-30. When the distance is smaller than 4.5 Å, the ligand reached the binding pocket of SARS-CoV-2 Mpro.

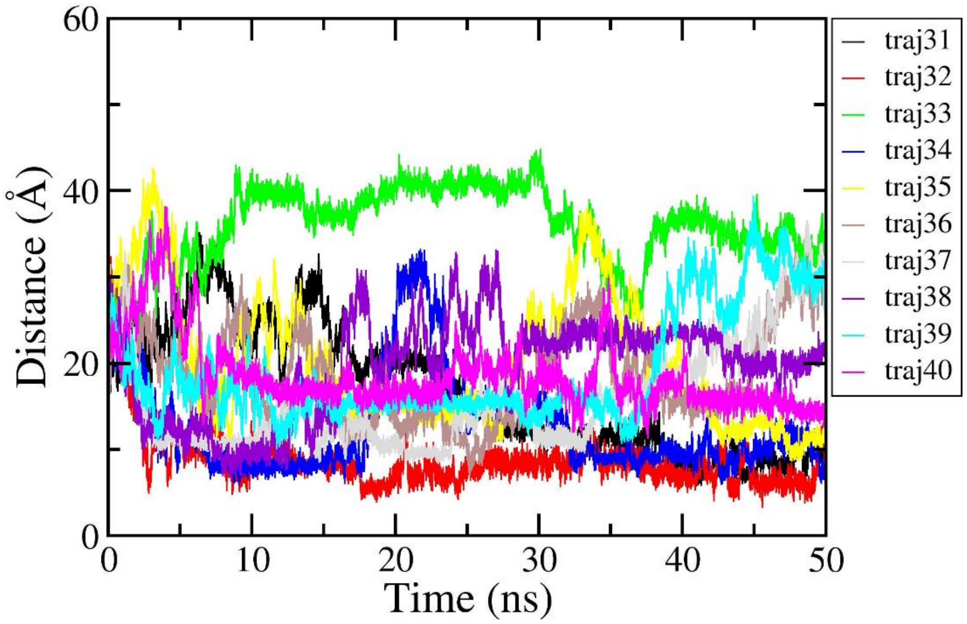

Figure S5. The minimum distance between non-hydrogen atoms of PF-07321332 and non-hydrogen atoms of the catalytic dyad (His41 and Cys145 residues). The results were obtained due to analyzing trajectories 31-40. When the distance is smaller than 4.5 Å, the ligand reached the binding pocket of SARS-CoV-2 Mpro.

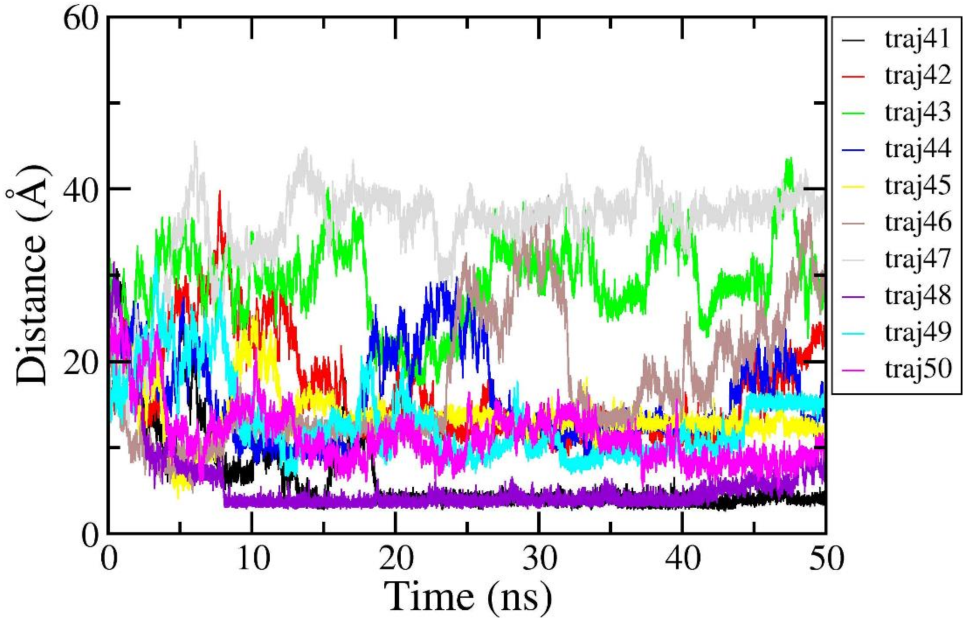

Figure S6. The minimum distance between non-hydrogen atoms of PF-07321332 and non-hydrogen atoms of the catalytic dyad (His41 and Cys145 residues). The results were obtained due to analyzing trajectories 41-50. When the distance is smaller than 4.5 Å, the ligand reached the binding pocket of SARS-CoV-2 Mpro.

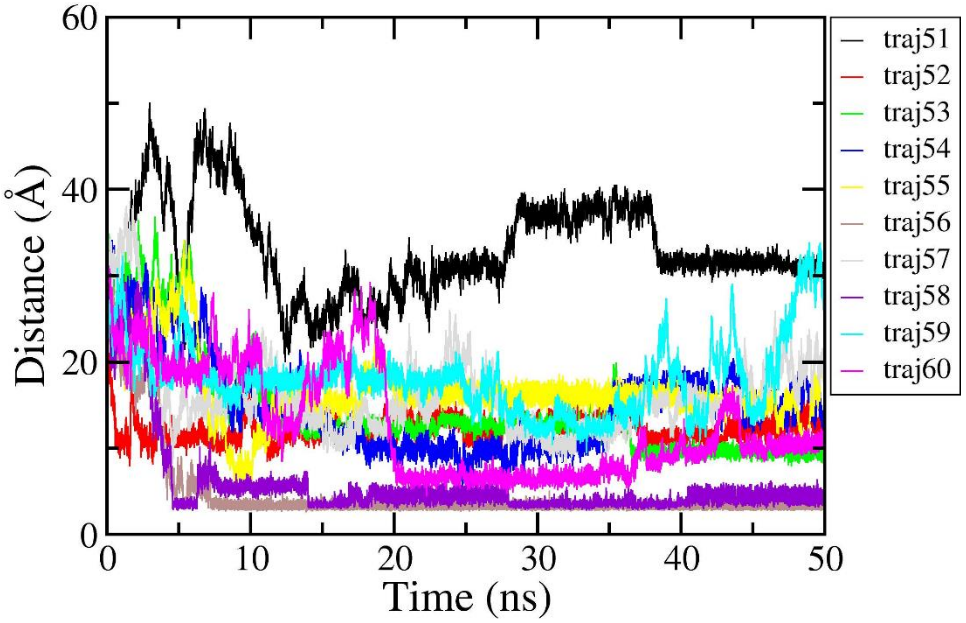

Figure S7. The minimum distance between non-hydrogen atoms of PF-07321332 and non-hydrogen atoms of the catalytic dyad (His41 and Cys145 residues). The results were obtained due to analyzing trajectories 51-60. When the distance is smaller than 4.5 Å, the ligand reached the binding pocket of SARS-CoV-2 Mpro.

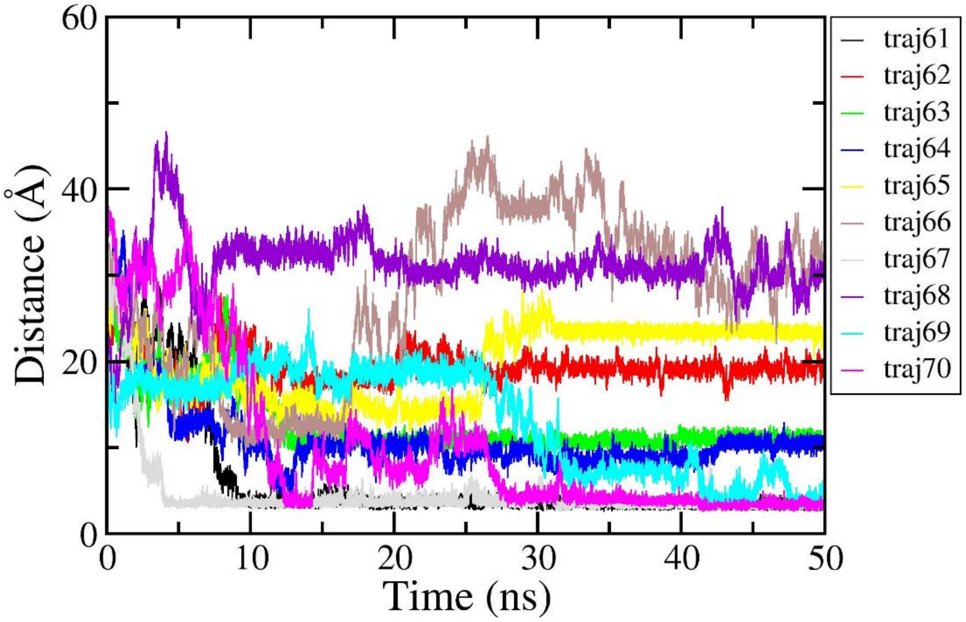

Figure S8. The minimum distance between non-hydrogen atoms of PF-07321332 and non-hydrogen atoms of the catalytic dyad (His41 and Cys145 residues). The results were obtained due to analyzing trajectories 61-70. When the distance is smaller than 4.5 Å, the ligand reached the binding pocket of SARS-CoV-2 Mpro.

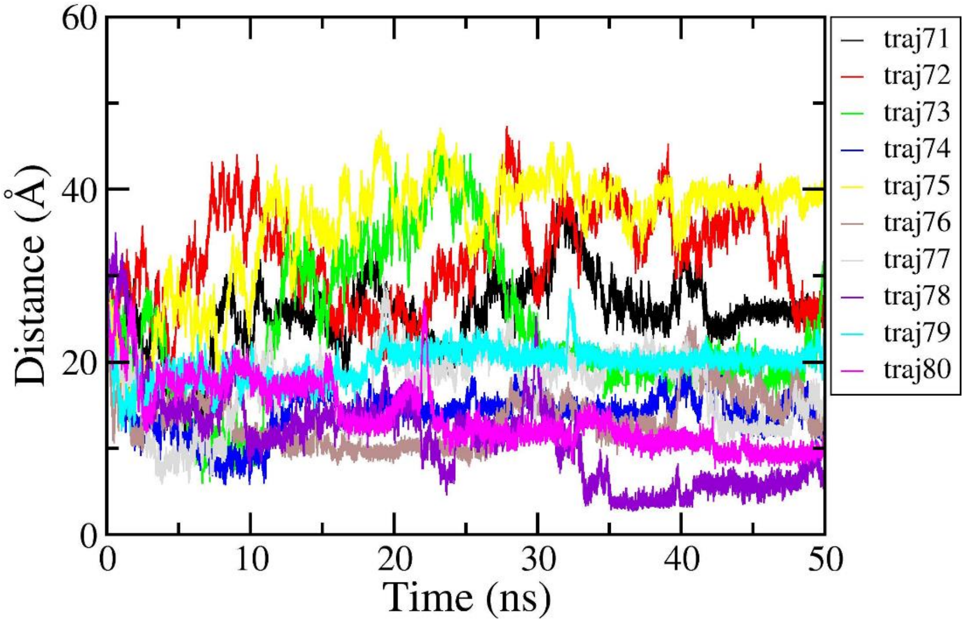

Figure S9. The minimum distance between non-hydrogen atoms of PF-07321332 and non-hydrogen atoms of the catalytic dyad (His41 and Cys145 residues). The results were obtained due to analyzing trajectories 71-80. When the distance is smaller than 4.5 Å, the ligand reached the binding pocket of SARS-CoV-2 Mpro.

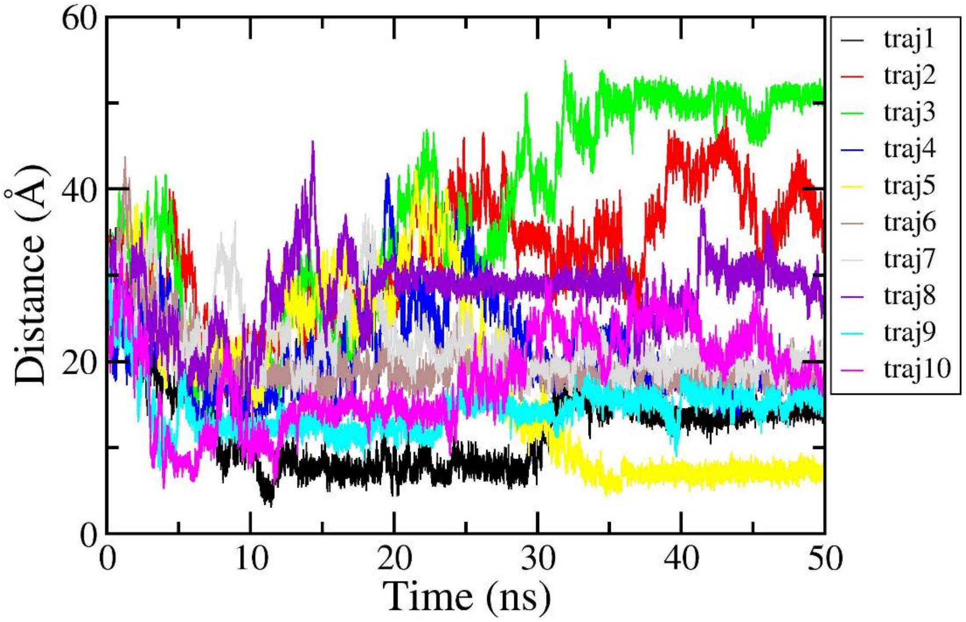

Figure S10. The minimum distance $d_{S\gamma-CN}$ between the nitrile group of PF-07321332 and the sulfur atom of the residue Cys145. The results were obtained due to analyzing trajectories 1-10. When the distance is smaller than 4.5 Å, the ligand reached the *bound* state.

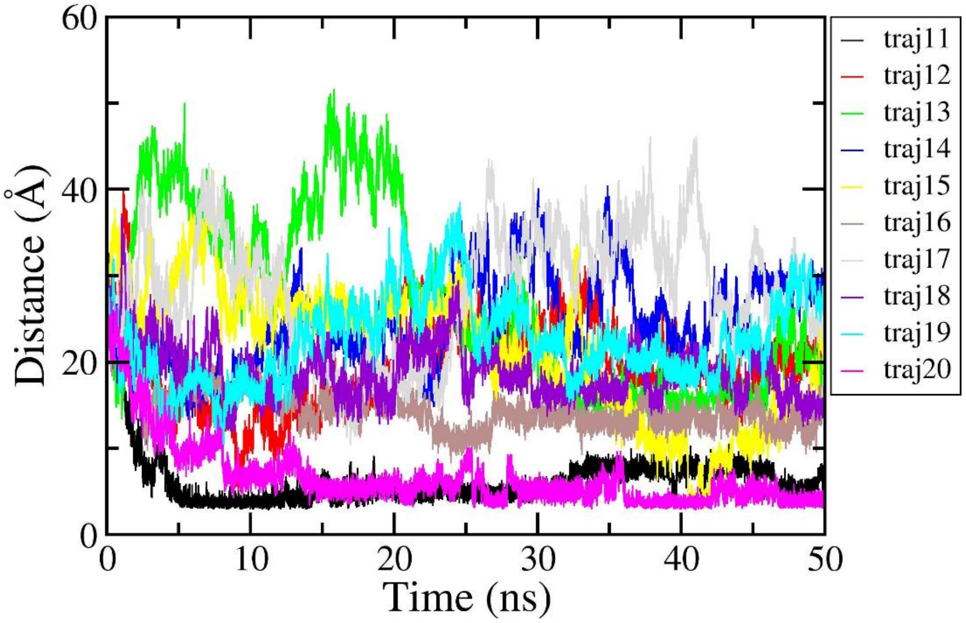

Figure S11. The minimum distance $d_{S\gamma-CN}$ between the nitrile group of PF-07321332 and the sulfur atom of the residue Cys145. The results were obtained due to analyzing trajectories 11-20. When the distance is smaller than 4.5 Å, the ligand reached the *bound* state.

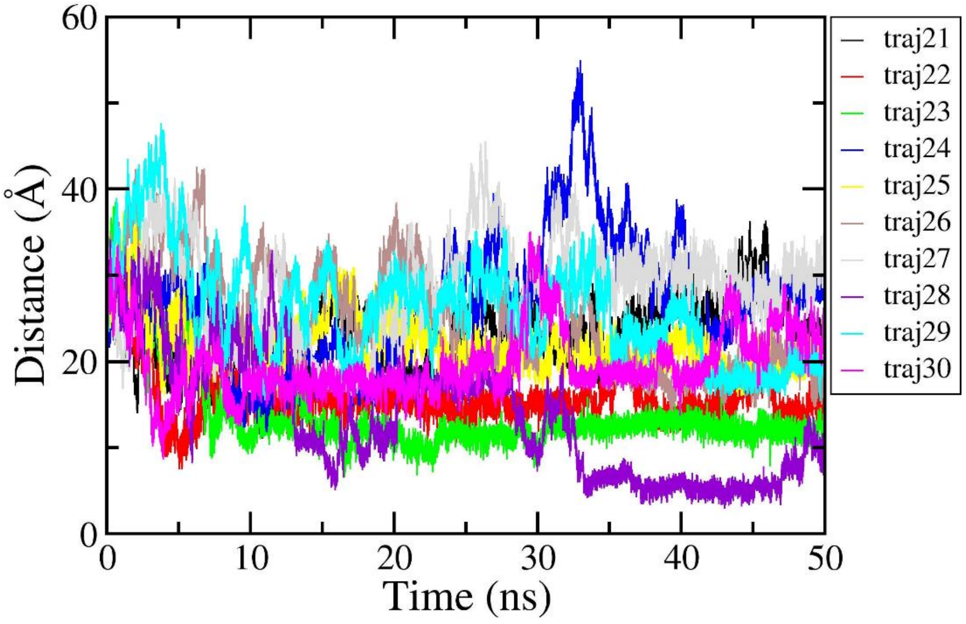

Figure S12. The minimum distance $d_{S\gamma-CN}$ between the nitrile group of PF-07321332 and the sulfur atom of the residue Cys145. The results were obtained due to analyzing trajectories 21-30. When the distance is smaller than 4.5 Å, the ligand reached the *bound* state.

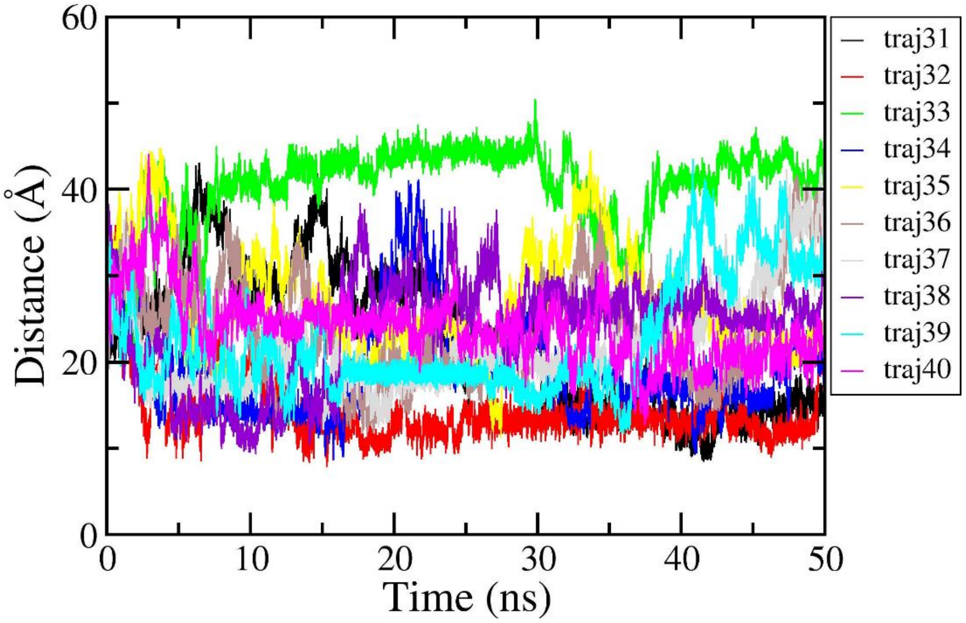

Figure S13. The minimum distance $d_{S\gamma-CN}$ between the nitrile group of PF-07321332 and the sulfur atom of the residue Cys145. The results were obtained due to analyzing trajectories 31-40. When the distance is smaller than 4.5 Å, the ligand reached the *bound* state.

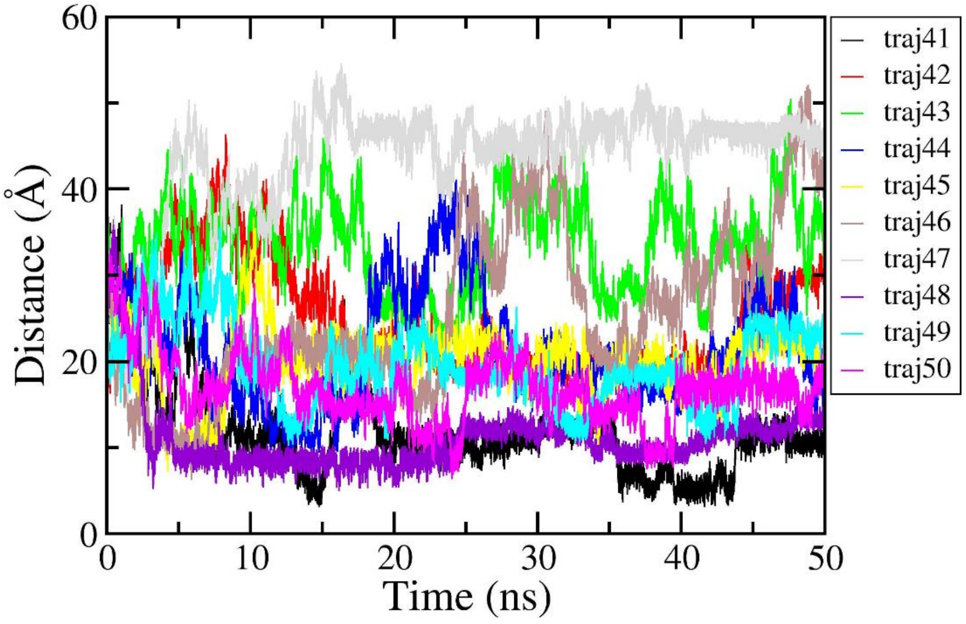

Figure S14. The minimum distance $d_{S\gamma-CN}$ between the nitrile group of PF-07321332 and the sulfur atom of the residue Cys145. The results were obtained due to analyzing trajectories 41-50. When the distance is smaller than 4.5 Å, the ligand reached the *bound* state.

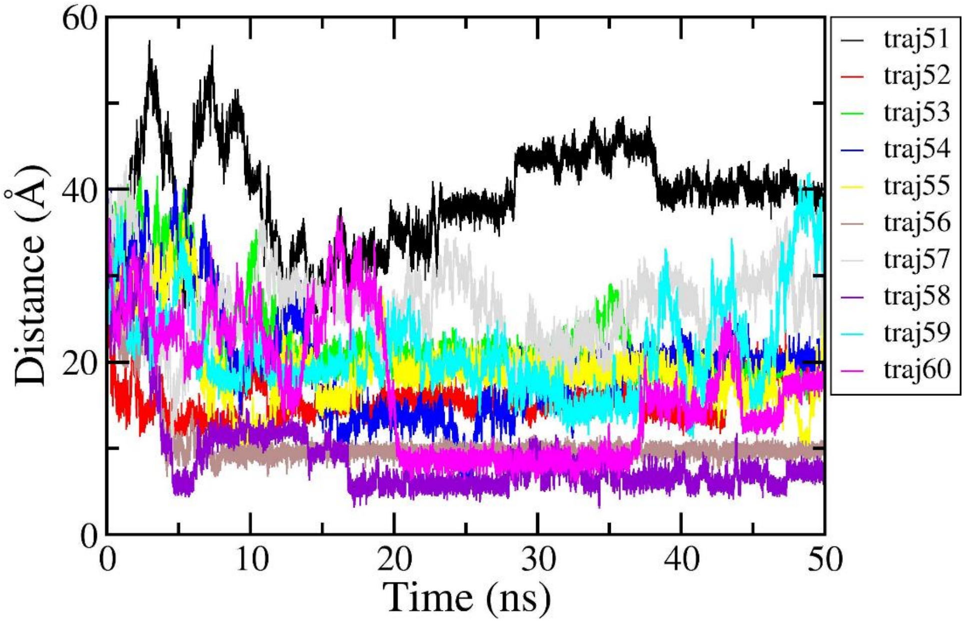

Figure S15. The minimum distance $d_{S\gamma-CN}$ between the nitrile group of PF-07321332 and the sulfur atom of the residue Cys145. The results were obtained due to analyzing trajectories 51-60. When the distance is smaller than 4.5 Å, the ligand reached the *bound* state.

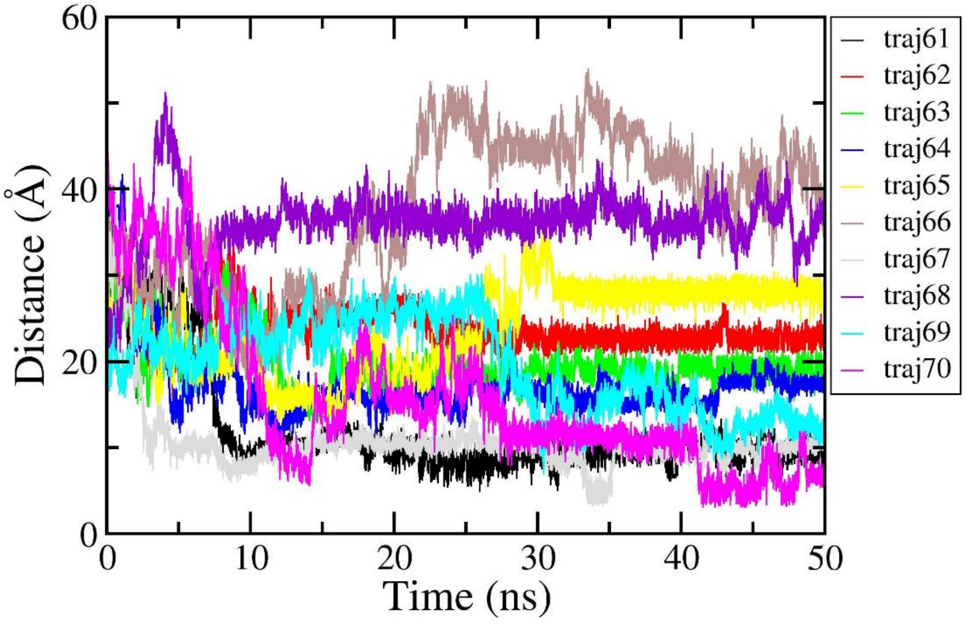

Figure S16. The minimum distance $d_{S\gamma-CN}$ between the nitrile group of PF-07321332 and the sulfur atom of the residue Cys145. The results were obtained due to analyzing trajectories 61-70. When the distance is smaller than 4.5 Å, the ligand reached the *bound* state.

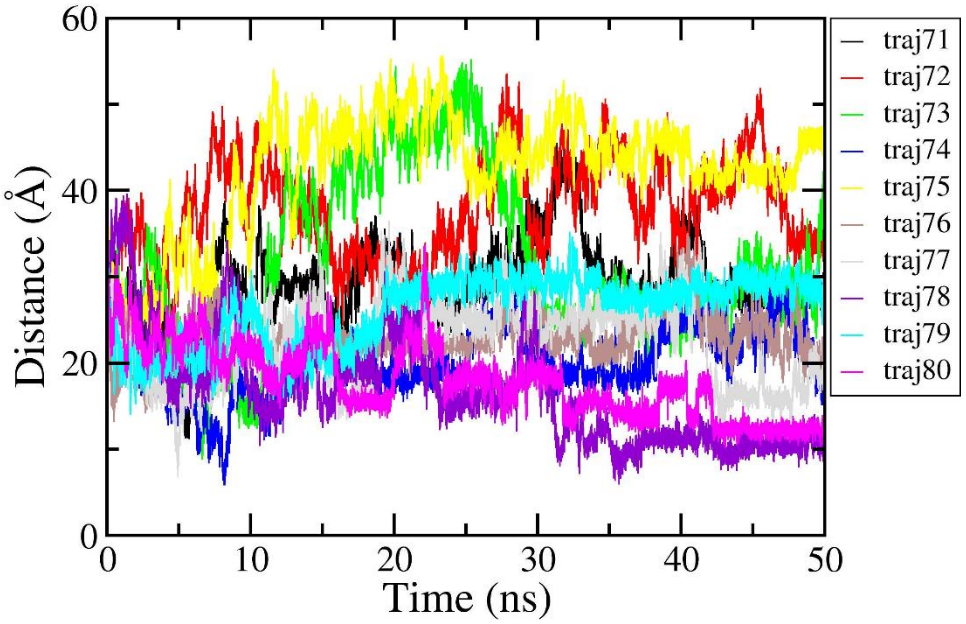

Figure S17. The minimum distance $d_{S\gamma-CN}$ between the nitrile group of PF-07321332 and the sulfur atom of the residue Cys145. The results were obtained due to analyzing trajectories 71-80. When the distance is smaller than 4.5 Å, the ligand reached the *bound* state.

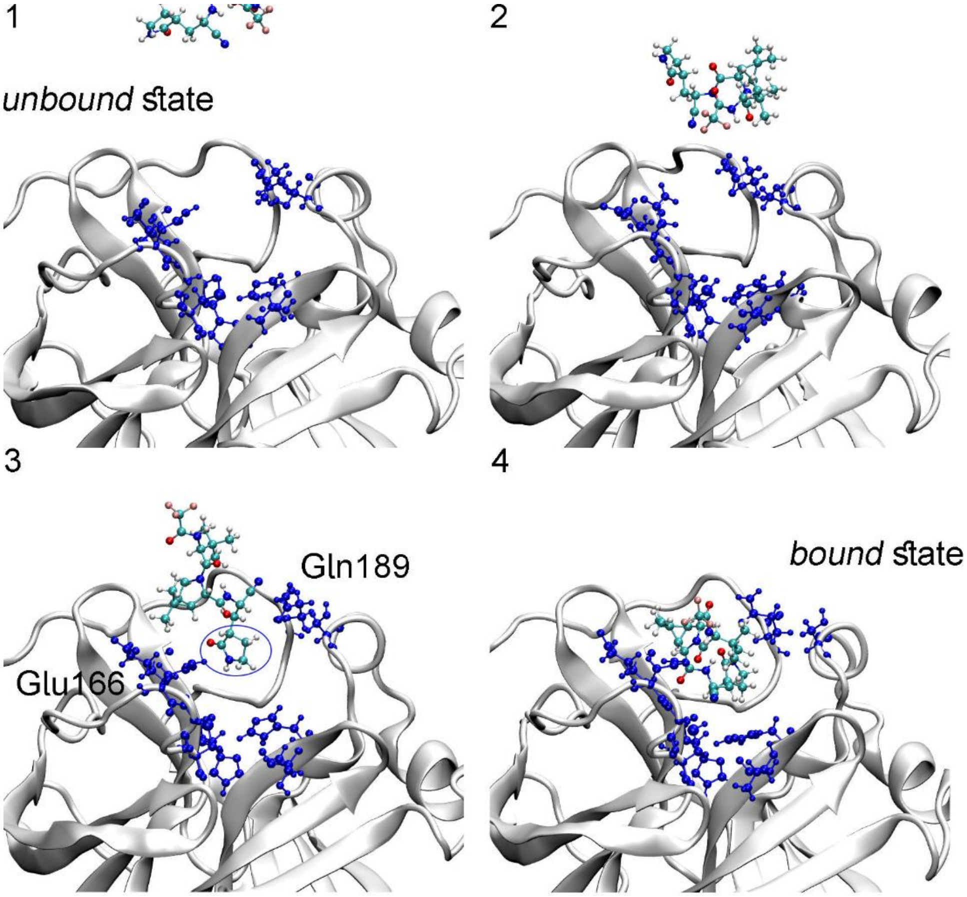

Figure S18. The binding pathway of PF-07321332 to SARS-CoV-2 Mpro over the trajectory 0. The results were obtained via the clustering analysis within a cutoff of 1.2 Å over the first 50 ns of the SMD trajectory. Two *unbound* clusters were not presented in the figure. The ligand mobilizes from *unbound* state **1** to *bound* state **4** with two *transition* states **2** and **3**. In particular, the pyrrolidinyl group of the ligand (noted with an ellipse) first inserted itself into the space between the residues Glu166 and Gln189, then the whole of the compound fully inserted into the binding pocket of SARS-CoV-2 Mpro.

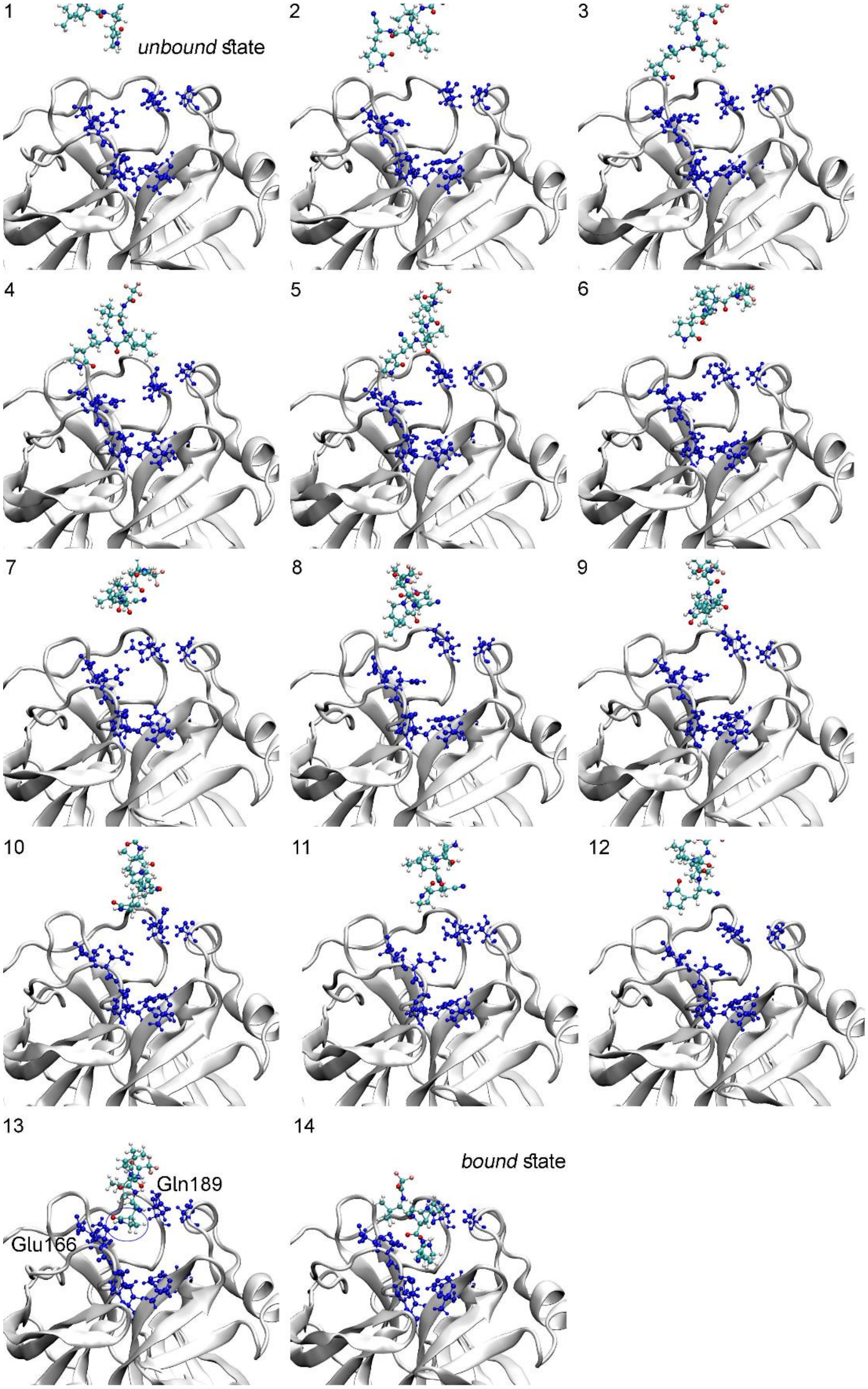

Figure S19. The binding pathway of PF-07321332 to SARS-CoV-2 Mpro over the trajectory 20. The results were obtained via the clustering analysis within a cutoff of 1.2 Å over the first 50 ns of the SMD trajectory. Two *unbound* clusters were not presented in the figure. The ligand mobilizes from *unbound* state **1** to

bound state **14** with twelve *transition* states from **2** to **13**. In particular, interestingly, the pyrrolidinyl group of the ligand (noted with an ellipse) first inserted itself into the space between the residues Glu166 and Gln189, then the whole of the compound fully inserted into the binding pocket of SARS-CoV-2 Mpro.

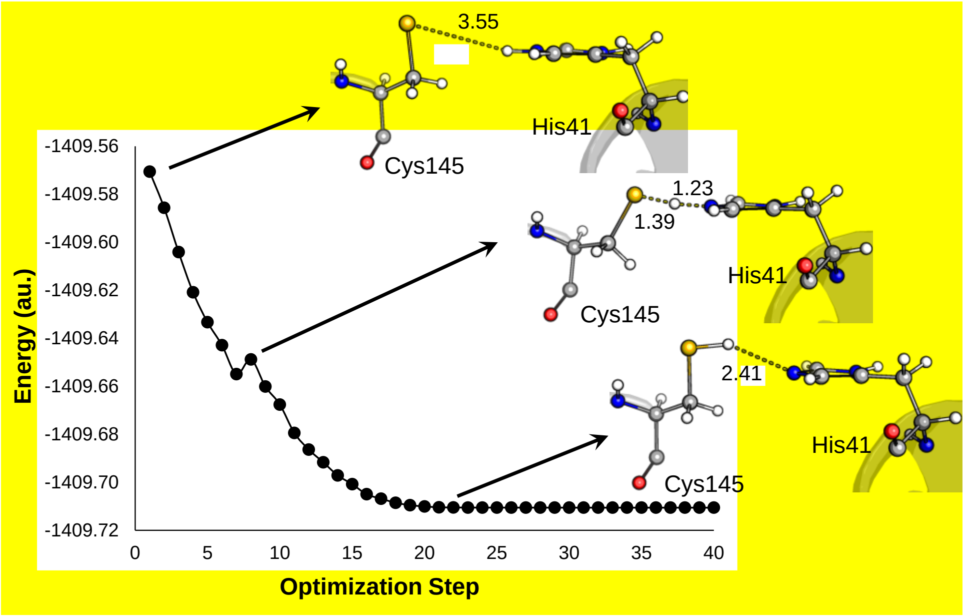

Figure S20. The optimization starting from ion-pair Cys145⁻–His41H⁺.

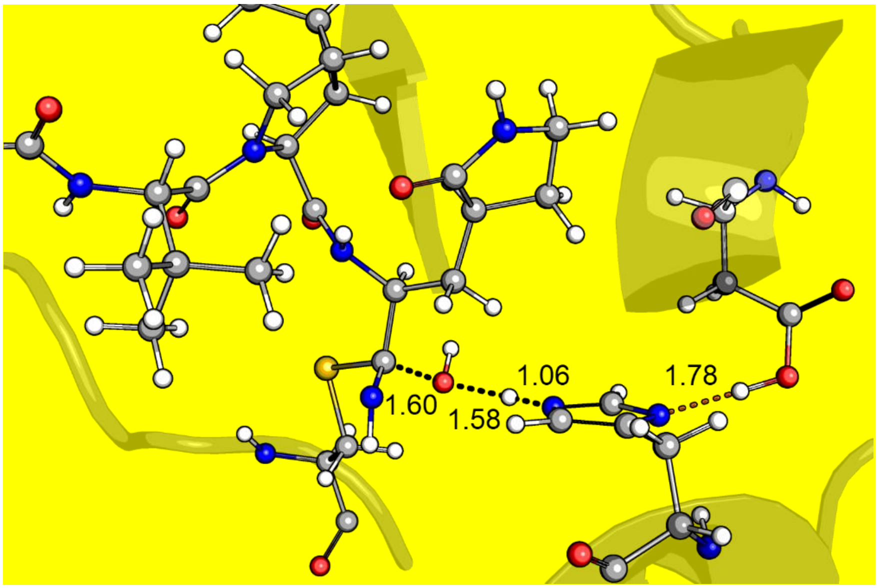

Figure S21. Optimized transition state for the hydrolysis of **Int-3**.

Energy and Cartesian Coordinate

Int-1

B3LYP-D3(BJ) SCF energy (au)	-1638.23497615
B3LYP-D3(BJ) enthalpy (au)	-1629.55549115
B3LYP-D3(BJ) free energy (au)	-1630.61997015
M06-2X SCF energy (au)	-1638.05661166
M06-2X enthalpy (au)	-1629.37712666
M06-2X free energy (au)	-1630.44160566

Cartesian coordinates

ATOM	X	Y	Z
C	70.915329	42.942291	41.548508
H	71.228363	42.893314	42.598576
H	70.675339	43.990105	41.329891
C	69.678757	42.129425	41.340706
N	68.694824	42.536613	40.463619
H	68.686905	43.484825	39.961918
C	67.693459	41.623493	40.548470
H	66.774841	41.718979	39.989101
N	67.982643	40.623184	41.373798
C	69.227325	40.942966	41.883255
H	69.715965	40.310196	42.613876
C	66.642220	36.912037	41.234039
H	67.545242	36.413811	41.585087
H	66.899879	37.592152	40.419720
S	65.870216	37.888058	42.585045
H	66.484421	39.064880	42.267178
C	66.941711	45.382915	40.573418
H	67.248413	44.487537	41.125359
H	66.132813	45.060623	39.903893
C	68.120125	45.892696	39.695503
O	68.169136	47.093033	39.353413
O	68.940231	44.986633	39.311420
C	67.540733	38.865330	45.598957
N	68.335243	38.025715	45.703857
O	63.896214	39.269794	46.322742
C	64.624931	39.931084	47.045002
N	65.938126	40.202740	46.801746
O	67.174049	42.644680	47.564034
N	66.442589	44.489296	46.390774
C	66.732101	43.171116	46.542465
C	66.555832	39.961288	45.506123
C	67.220200	41.238346	44.923351
C	66.384560	42.485920	45.213081
C	66.459183	43.653732	44.216679
C	65.951057	44.842022	45.060349
H	66.404434	40.865200	47.422573
H	66.367432	45.089481	47.209846

H	65.749779	39.632214	44.842693
H	67.340714	41.096527	43.845009
H	68.220406	41.360947	45.349812
H	65.328468	42.193649	45.303959
H	65.869080	43.486660	43.313927
H	67.497108	43.825352	43.911865
H	64.852966	44.902805	45.046555
H	66.349709	45.807125	44.742680

TS-1

B3LYP-D3(BJ) SCF energy (au)	-1638.23093803
B3LYP-D3(BJ) enthalpy (au)	-1629.55622703
B3LYP-D3(BJ) free energy (au)	-1630.61713203
M06-2X SCF energy (au)	-1638.05430941
M06-2X enthalpy (au)	-1629.37959841
M06-2X free energy (au)	-1630.44050341

Cartesian coordinates

ATOM	X	Y	Z
C	70.969643	42.950951	41.585014
H	71.291237	42.854134	42.630474
H	70.772064	44.016636	41.413910
C	69.701714	42.190250	41.355331
N	68.733543	42.644718	40.482922
H	68.747581	43.812752	39.890305
C	67.740356	41.723606	40.551830
H	66.819588	41.823898	39.992638
N	68.007385	40.690407	41.357361
C	69.250114	40.990482	41.876266
H	69.742287	40.333595	42.584717
C	66.722511	36.895283	41.211250
H	67.579247	36.363644	41.624847
H	67.052086	37.503822	40.366528
S	65.970901	38.005955	42.461334
H	66.645363	39.140446	42.080364
C	66.878746	45.320534	40.607388
H	67.185684	44.430180	41.164429
H	66.074631	44.990955	39.937561
C	68.043159	45.820923	39.732777
O	68.117935	46.993328	39.342819
O	68.893906	44.904015	39.357639
C	67.319977	38.433636	45.661034
N	67.938965	37.463646	45.813503
O	63.852913	39.158970	46.323811
C	64.605568	39.797916	47.042152
N	65.921951	40.025169	46.797344
O	67.503883	42.094707	47.764759
N	67.424065	44.098694	46.653198

C	67.326736	42.752598	46.738625
C	66.564308	39.698277	45.536339
C	67.504135	40.832138	45.042965
C	66.959564	42.232903	45.340504
C	67.464340	43.370445	44.430687
C	67.303101	44.636944	45.304989
H	66.441414	40.612991	47.452286
H	67.506958	44.648624	47.506130
H	65.764458	39.537815	44.806835
H	67.640472	40.711445	43.964542
H	68.487831	40.724995	45.511185
H	65.860519	42.212742	45.301670
H	66.920929	43.434795	43.486645
H	68.519638	43.212021	44.185692
H	66.328194	45.120365	45.153915
H	68.086113	45.377975	45.128632

Int-2

B3LYP-D3(BJ) SCF energy (au)	-1638.23353145
B3LYP-D3(BJ) enthalpy (au)	-1629.55468145
B3LYP-D3(BJ) free energy (au)	-1630.61802645
M06-2X SCF energy (au)	-1638.05519724
M06-2X enthalpy (au)	-1629.37634724
M06-2X free energy (au)	-1630.43969224

Cartesian coordinates

ATOM	X	Y	Z
C	70.966667	42.921268	41.602394
H	71.293861	42.813686	42.645752
H	70.774429	43.991482	41.448547
C	69.692726	42.168537	41.370636
N	68.728394	42.628155	40.495384
H	68.772797	44.098324	39.725163
C	67.745544	41.699013	40.578274
H	66.820633	41.783222	40.020012
N	68.009308	40.662544	41.390724
C	69.251358	40.967979	41.904804
H	69.747925	40.315849	42.615582
C	66.753296	36.892654	41.203617
H	67.597519	36.346573	41.624775
H	67.099831	37.483597	40.352852
S	66.024940	38.036945	42.435947
H	66.734589	39.157406	42.045708
C	66.897881	45.353340	40.585609
H	67.258995	44.466995	41.115566
H	66.097702	44.998947	39.924450
C	68.008774	45.919151	39.705299
O	68.073723	47.085403	39.327213

O 68.898979 45.033211 39.274395
C 67.313866 38.407356 45.673534
N 67.932854 37.439304 45.837807
O 63.843307 39.148808 46.328403
C 64.599960 39.786957 47.043419
N 65.916725 40.007843 46.797089
O 67.504890 42.076008 47.758587
N 67.439003 44.074272 46.637127
C 67.333687 42.728664 46.728230
C 66.560173 39.671947 45.538342
C 67.503922 40.800068 45.039669
C 66.966553 42.204487 45.332176
C 67.481613 43.333241 44.416874
C 67.336098 44.606426 45.285217
H 66.438240 40.595318 47.450520
H 67.531441 44.626553 47.487431
H 65.762054 39.507915 44.807884
H 67.638191 40.676563 43.961201
H 68.488144 40.690884 45.506294
H 65.867470 42.190594 45.290897
H 66.936470 43.395592 43.473637
H 68.533440 43.160564 44.167343
H 66.370834 45.106739 45.126160
H 68.133560 45.332787 45.110210

TS-2

B3LYP-D3(BJ) SCF energy (au)	-1638.22885522
B3LYP-D3(BJ) enthalpy (au)	-1629.55304822
B3LYP-D3(BJ) free energy (au)	-1630.61495122
M06-2X SCF energy (au)	-1638.05015893
M06-2X enthalpy (au)	-1629.37435193
M06-2X free energy (au)	-1630.43625493

Cartesian coordinates

ATOM X Y Z
C 70.879265 42.815006 41.612267
H 71.186348 42.692822 42.658928
H 70.661331 43.882294 41.476891
C 69.627472 42.038841 41.341454
N 68.661873 42.509525 40.467579
H 68.727951 44.054901 39.688564
C 67.688248 41.582645 40.510777
H 66.761581 41.656433 39.959167
N 67.977531 40.532314 41.301075
C 69.206604 40.823532 41.848080
H 69.680473 40.155773 42.555775
C 66.845787 36.957184 41.166805
H 67.617950 36.378513 41.679726

H 67.294983 37.401714 40.271793
S 66.200859 38.303528 42.235378
H 67.161270 39.519325 41.699863
C 66.907944 45.321491 40.616329
H 67.274170 44.440052 41.151012
H 66.098938 44.961735 39.968521
C 68.006813 45.879219 39.719582
O 68.091888 47.045128 39.351898
O 68.877502 44.979675 39.263584
C 67.472054 38.473579 45.605633
N 68.209763 37.593300 45.773849
O 63.838028 39.091908 46.294422
C 64.608368 39.708084 47.012707
N 65.931610 39.904930 46.766525
O 67.377365 42.202682 47.591335
N 66.802574 44.129921 46.471401
C 66.978088 42.787643 46.585632
C 66.554649 39.623402 45.480831
C 67.307060 40.862656 44.920719
C 66.574562 42.168121 45.239601
C 66.752876 43.354355 44.274708
C 66.349998 44.560108 45.152470
H 66.440063 40.512489 47.408298
H 66.825371 44.717609 47.303104
H 65.759262 39.334270 44.787041
H 67.405411 40.733250 43.840961
H 68.317757 40.905346 45.338917
H 65.496773 41.963848 45.315536
H 66.146324 43.260113 43.371647
H 67.801689 43.441807 43.969337
H 65.261673 44.720028 45.137001
H 66.834709 45.495827 44.868496

Int-3

B3LYP-D3(BJ) SCF energy (au)	-1638.23304493
B3LYP-D3(BJ) enthalpy (au)	-1629.55188193
B3LYP-D3(BJ) free energy (au)	-1630.61433293
M06-2X SCF energy (au)	-1638.05534309
M06-2X enthalpy (au)	-1629.37418009
M06-2X free energy (au)	-1630.43663109

Cartesian coordinates
ATOM X Y Z
C 70.955132 42.903286 41.571533
H 71.260788 42.786293 42.618317
H 70.754951 43.971687 41.422470
C 69.692467 42.145618 41.307644
N 68.744743 42.612335 40.408413

H	68.797737	44.212200	39.624233
C	67.755760	41.714970	40.446285
H	66.831970	41.780106	39.891865
N	68.035233	40.682667	41.266384
C	69.256721	40.950035	41.837666
H	69.694794	40.280144	42.563091
C	66.842674	36.936157	41.194614
H	67.598824	36.344307	41.719852
H	67.316910	37.341206	40.290897
S	66.228546	38.318371	42.242359
H	67.376968	39.856678	41.550983
C	66.940643	45.389267	40.584576
H	67.331573	44.509705	41.103470
H	66.140724	45.020981	39.930332
C	68.018761	45.999821	39.699802
O	68.071060	47.171127	39.352489
O	68.921387	45.138527	39.221668
C	67.505386	38.415985	45.649250
N	68.268044	37.563263	45.846600
O	63.824043	39.063576	46.305065
C	64.605148	39.663956	47.024078
N	65.933357	39.833195	46.782890
O	67.389229	42.147018	47.579494
N	66.780037	44.061684	46.455791
C	66.970291	42.720451	46.576092
C	66.560944	39.540440	45.501503
C	67.290756	40.784798	44.920486
C	66.554863	42.088394	45.238842
C	66.715126	43.270950	44.265621
C	66.310036	44.478939	45.139179
H	66.445793	40.433758	47.426823
H	66.811081	44.657185	47.281574
H	65.778435	39.220310	44.805470
H	67.363670	40.633739	43.842258
H	68.309090	40.841679	45.318863
H	65.479370	41.878342	45.326382
H	66.100449	43.167488	43.368816
H	67.761246	43.366909	43.952354
H	65.220078	44.628025	45.132629
H	66.782715	45.417320	44.842545

TS-3

B3LYP-D3(BJ) SCF energy (au)	-1714.64906809
B3LYP-D3(BJ) enthalpy (au)	-1705.94374809
B3LYP-D3(BJ) free energy (au)	-1707.00735809
M06-2X SCF energy (au)	-1714.47313992
M06-2X enthalpy (au)	-1705.76781992
M06-2X free energy (au)	-1706.83142992

Cartesian coordinates

ATOM	X	Y	Z
C	71.314636	43.233864	41.408207
H	71.572327	43.038780	42.455410
H	71.295097	44.323109	41.281998
C	69.968491	42.681591	41.065533
N	69.194984	43.240181	40.054115
H	69.134857	44.847050	39.455044
C	68.165154	42.407101	39.902958
H	67.344109	42.554665	39.215794
N	68.244408	41.349934	40.729900
C	69.379097	41.510441	41.486465
H	69.638443	40.779007	42.239029
C	66.589958	36.308701	42.304485
H	67.376488	35.679211	42.726898
H	67.020287	37.046852	41.629940
S	65.695648	37.184879	43.659088
H	67.674873	40.414845	40.722038
C	67.110878	45.652058	40.504322
H	67.632637	44.809139	40.960365
H	66.364090	45.213234	39.829563
C	68.065216	46.475262	39.649849
O	67.927925	47.657291	39.373360
O	69.078796	45.804848	39.098309
C	66.936378	38.659962	43.875938
N	67.897369	38.874023	43.120361
O	63.987579	38.756958	46.023449
C	64.868553	39.270599	46.696877
N	66.145447	39.487701	46.266479
O	67.749725	41.694355	47.534401
N	67.375427	43.880421	46.890877
C	67.338135	42.525482	46.730251
C	66.384323	39.747208	44.833790
C	67.238472	41.013687	44.658859
C	66.685989	42.247314	45.376209
C	66.861671	43.588360	44.642029
C	66.813278	44.631516	45.775383
H	66.739227	40.004017	46.915863
H	67.523628	44.286701	47.813335
H	65.388245	39.955460	44.427315
H	67.300064	41.210041	43.587929
H	68.265030	40.814957	44.981606
H	65.615913	42.106140	45.592503
H	66.103828	43.763744	43.875523
H	67.844513	43.612740	44.156761
H	65.782867	44.954613	45.986847
H	67.413109	45.521461	45.564629

O	67.109093	39.022205	40.798870
H	67.668999	38.758923	41.886559
H	66.258095	39.269466	41.234955

Int-4

B3LYP-D3(BJ) SCF energy (au)	-1714.69028153
B3LYP-D3(BJ) enthalpy (au)	-1705.97948053
B3LYP-D3(BJ) free energy (au)	-1707.04677453
M06-2X SCF energy (au)	-1714.51954544
M06-2X enthalpy (au)	-1705.80874444
M06-2X free energy (au)	-1706.87603844

Cartesian coordinates

ATOM	X	Y	Z
C	71.416008	43.328289	41.379482
H	71.742332	43.229843	42.422745
H	71.375496	44.403027	41.160950
C	70.057961	42.736832	41.181324
N	69.193039	43.190903	40.200695
H	69.123634	44.700809	39.520779
C	68.153542	42.329041	40.262596
H	67.273178	42.420567	39.635136
N	68.290276	41.352112	41.170181
C	69.502548	41.612312	41.763687
H	69.898727	40.975052	42.546631
C	66.643051	36.272724	42.321838
H	67.373795	35.593407	42.771317
H	67.109604	37.060406	41.719753
S	65.632645	37.113792	43.617935
H	68.394386	37.034039	44.013775
C	67.068886	45.601994	40.450546
H	67.537758	44.731308	40.912369
H	66.309906	45.205948	39.764011
C	68.084618	46.373806	39.618008
O	67.983574	47.557522	39.317940
O	69.095200	45.666077	39.124798
C	66.899666	38.168087	44.375885
N	68.145676	37.930138	44.437805
O	63.920540	38.668640	46.103443
C	64.764343	39.261833	46.763786
N	66.043549	39.478924	46.361710
O	67.651283	41.571575	47.447582
N	67.419525	43.704922	46.616219
C	67.367104	42.350922	46.538536
C	66.364197	39.499508	44.930485
C	67.345039	40.639721	44.601933
C	66.874168	42.000835	45.128567
C	67.301437	43.245705	44.326176

C	67.174255	44.393600	45.353909
H	66.634171	40.045120	46.975140
H	67.562889	44.174313	47.507839
H	65.416969	39.697277	44.426289
H	67.445313	40.685707	43.512833
H	68.333046	40.395340	45.000549
H	65.774811	42.000439	45.200680
H	66.697372	43.406841	43.431705
H	68.340851	43.139645	43.997459
H	66.174118	44.850281	45.338276
H	67.913834	45.185249	45.206921
O	66.974304	38.951344	41.018467
H	67.532860	39.781742	41.073582
H	66.205460	39.248974	41.558372

TS-4

B3LYP-D3(BJ) SCF energy (au)	-1714.63270762
B3LYP-D3(BJ) enthalpy (au)	-1705.92355662
B3LYP-D3(BJ) free energy (au)	-1706.98846362
M06-2X SCF energy (au)	-1714.45721790
M06-2X enthalpy (au)	-1705.74806690
M06-2X free energy (au)	-1706.81297390

Cartesian coordinates

ATOM	X	Y	Z
C	71.378471	43.329632	41.386139
H	71.701477	43.260509	42.431103
H	71.286156	44.395943	41.147930
C	70.051796	42.673180	41.194397
N	69.168625	43.084007	40.202785
H	69.081375	44.712410	39.489670
C	68.143036	42.234329	40.274429
H	67.243690	42.284966	39.674461
N	68.343178	41.289787	41.214775
C	69.541771	41.561649	41.824635
H	69.891457	40.943295	42.639576
C	66.469124	36.558502	41.952625
H	67.407631	36.071705	42.229660
H	66.691185	37.384991	41.274094
S	65.617874	37.237309	43.440247
H	68.664467	37.929394	43.284588
C	67.083275	45.593975	40.523846
H	67.575760	44.741402	40.995373
H	66.323242	45.172337	39.852943
C	68.067726	46.372169	39.663094
O	67.970016	47.552589	39.368526
O	69.066589	45.661476	39.126808
C	67.028862	38.836533	43.698917

N	68.268837	38.561596	43.982742
O	63.947559	38.891720	45.827946
C	64.862373	39.372383	46.479679
N	66.123131	39.600113	46.010216
O	67.699959	41.894485	47.330112
N	67.141029	44.057907	46.736259
C	67.195770	42.701466	46.558620
C	66.332939	39.902920	44.584713
C	67.126091	41.215523	44.475716
C	66.504639	42.390938	45.231800
C	66.523888	43.751507	44.517204
C	66.440414	44.766724	45.672817
H	66.751335	40.065361	46.662483
H	67.285797	44.455963	47.663078
H	65.325363	40.050049	44.190941
H	67.193466	41.475193	43.420605
H	68.145599	41.025127	44.822544
H	65.463364	42.152645	45.494877
H	65.715164	43.869682	43.791988
H	67.477425	43.869305	43.989414
H	65.396164	44.979767	45.948196
H	66.932777	45.715984	45.444298
O	66.812393	39.387726	42.214821
H	67.687889	40.530567	41.571617
H	65.893867	39.748531	42.199726
